# Sustained IFN-I Expression during Established Persistent Viral Infection: A “Bad Seed” for Protective Immunity

**DOI:** 10.3390/v10010012

**Published:** 2017-12-30

**Authors:** Xavier Dagenais-Lussier, Hamza Loucif, Armstrong Murira, Xavier Laulhé, Simona Stäger, Alain Lamarre, Julien van Grevenynghe

**Affiliations:** Institut National de la Recherche Scientifique (INRS)-Institut Armand-Frappier, 531 Boulevard des Prairies, Laval, H7V 1B7 QC, Canada; xavier.dagenais@iaf.inrs.ca (X.D.-L.); hamza.loucif@iaf.inrs.ca (H.L.); armstrong.murira@iaf.inrs.ca (A.M.); xavier.laulhe@iaf.inrs.ca (X.L.); simona.stager@iaf.inrs.ca (S.S.); alain.lamarre@iaf.inrs.ca (A.L.)

**Keywords:** sustained IFN-I expression, IFNR blockade, persistent infection, exhaustion, immune activation/inflammation, immunosuppression, cell loss

## Abstract

Type I interferons (IFN-I) are one of the primary immune defenses against viruses. Similar to all other molecular mechanisms that are central to eliciting protective immune responses, IFN-I expression is subject to homeostatic controls that regulate cytokine levels upon clearing the infection. However, in the case of established persistent viral infection, sustained elevation of IFN-I expression bears deleterious effects to the host and is today considered as the major driver of inflammation and immunosuppression. In fact, numerous emerging studies place sustained IFN-I expression as a common nexus in the pathogenesis of multiple chronic diseases including persistent infections with the human immunodeficiency virus type 1 (HIV-1), simian immunodeficiency virus (SIV), as well as the rodent-borne lymphocytic choriomeningitis virus clone 13 (LCMV clone 13). In this review, we highlight recent studies illustrating the molecular dysregulation and resultant cellular dysfunction in both innate and adaptive immune responses driven by sustained IFN-I expression. Here, we place particular emphasis on the efficacy of IFN-I receptor (IFNR) blockade towards improving immune responses against viral infections given the emerging therapeutic approach of blocking IFNR using neutralizing antibodies (Abs) in chronically infected patients.

## 1. IFN-I and Its Beneficial Role

The type I interferon (IFN-I) system consists of five types of interferon: IFN-α, IFN-β, IFN-ω, IFN-ε, and IFN-κ. This is based on the structure of their respective receptors on the cell surface. Of these, only IFN-α is encoded by more than a single gene (13 subtypes in humans, 14 in mice). Once they bind with their receptors, they trigger the downstream induction of interferon-stimulated genes (ISGs) through the Janus kinase/signal transducers and activators of transcription signaling pathway. These ISGs include various intrinsic restriction factors, cytokines, chemokines, and co-stimulatory molecules in infected cells as well as bystander uninfected cells, all of which have been largely reviewed elsewhere [1,2,3]. IFN-I is considered to be the most potent autocrine and paracrine secreted “virus-induced” cytokine and is critical in establishing an efficient adaptive and acquired immune response especially in acute infections [4]. In the context of chronic infections, many studies showed a deficiency in IFN-I induced-antiviral responses. This lead to the use of IFN-I as treatment for simian immunodeficiency virus (SIV), hepatitis C virus (HCV), and hepatitis B virus (HBV) infections which demonstrates its positive, although moderate, effect during the acute phase of chronic infections [2,5,6]. However, cluster of differentiation 8 (CD8) T-cell hyperactivation has been observed in HCV-infected patients that are continuously treated with IFN-I over seven months [7,8]. Considering that IFN-I signaling is crucial in controlling viral infections, the focus of this review seems counterintuitive, yet it is now well established that sustained IFN-I expression also contributes in the establishment of a persistent infection escalated by immune hyperactivation and inflammation [1,3,7].

## 2. Sustained IFN-I Expression Drives Disease Progression during Persistent Infection

Persistent viral infections, including human immunodeficiency virus type 1 (HIV-1), are associated with prolonged dysregulation of multiple key signaling pathways, such as programmed cell death protein 1 (PD-1) and transcription factor forkhead Box O3 (FOXO3a), whose functions are critical in controlling and eliciting effective immune responses. Conversely, in the face of chronic inflammation and viral persistence, these signaling pathways drive immune dysfunction that ultimately contributes to disease progression. Importantly, these aberrancies are reversible as illustrated by the improvement in related protective immunity upon targeted blockade of the exhaustion marker, PD-1, and transcription factor, FOXO3a. Of note, both of these pathways have been found to be overexpressed in chronically HIV-1-infected patients despite induction of antiretroviral therapy (ART) wherein immune function is rescued by blockade even after years of infection [9,10,11,12,13,14]. To date, there are well-established lines of evidence placing sustained IFN-I expression as a major contributor of immune activation/inflammation and resultant disease progression during persistent viral infections such as HIV-1 and SIV [1,3,6,7,8,15,16,17,18,19,20,21,22,23,24,25,26,27,28,29]. For example, in the non-human primate model of SIV infection, natural hosts that do not progress toward acquired immune deficiency syndrome (AIDS) exhibit lower levels of IFN-I signaling and inflammation when compared to animals harbouring pathogenic infections [6,30,31,32,33]. In the context of HIV-1 infection, disease severity, reduction of CD4 T-cell counts, and poor immune restoration after ART are associated with sustained and elevated IFN-I expression [8,20,34,35,36]. The maintenance of IFN-I expression in patients under ART is due to the persistence of residual viral replication in tissues and local inflammation [37]. Interestingly, in vivo blockade of the interferon-α/β receptor (IFNR) during persistent HIV-1 infection, which reversed HIV-1-induced immune hyperactivation, also rescued anti-HIV-1 immune responses and induced the reactivation of viral reservoir in the presence of ART. More importantly, IFNR blockade reduced cell-associated virus numbers and significantly controlled HIV-1 rebound after ART cessation [18,38]. Thus, it is unsurprising that the use of humanized anti-IFNR blocking antibodies (Abs), which are currently used in a phase I trial in systemic lupus erythematosus and systemic sclerosis [39,40], are now proposed to treat persistent viral infections as a means of reducing chronic inflammation [3,27,41].

## 3. Impact of Sustained IFN-I Expression on the Innate Immune Response

**Figure 1:**
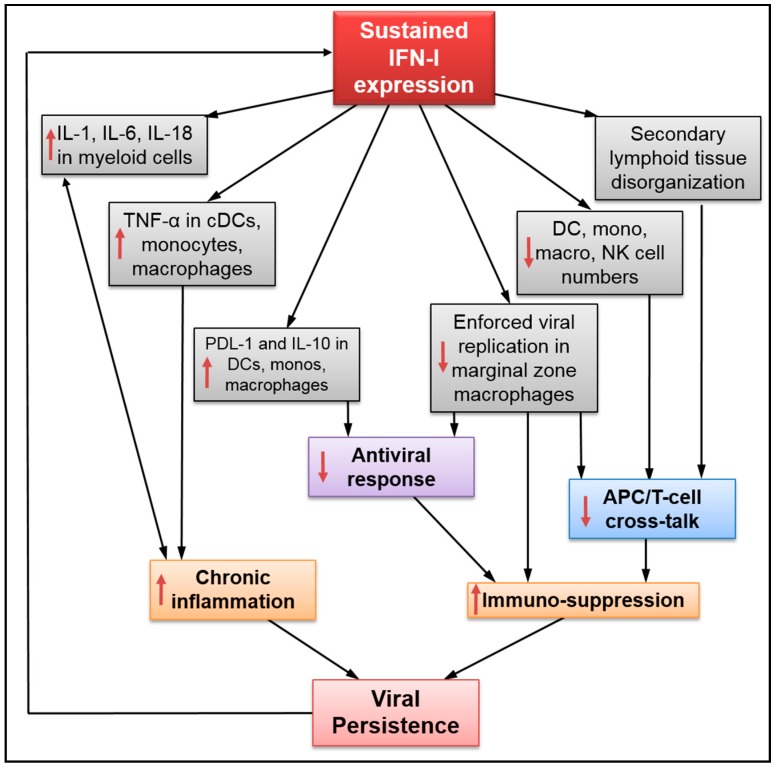
Defects mediated by sustained type I interferon (IFN-I) expression on innate immunity during persistent viral infection. APC: antigen presenting cells; TNF: tumor necrosis factor; DC: dendritic cell; cDCs: conventional dendritic cell; PDL: programmed death ligand; NK: natural killer; ↑: up-regulation; ↓: down-regulation.

Upon establishment of persistent viral infection, IFN-I is mainly produced by activated plasmacytoid dendritic cells (pDCs) and to a lesser extent by monocytes, macrophages, and conventional DCs (cDCs) by pathogen sensing [8,15,42,43]. A recent study has also shown that polyclonal Abs and Ab complexes found in HIV-1-infected subjects induced IFN-I production in pDCs [44]. As depicted in Figure 1, sustained IFN-I expression drives dysregulation at the tissue as well as cellular level within the innate immune response. For instance, IFN-I is shown to have a role in pDC loss during systemic viral infections. Using IFNR knockout mice, Swiecki and colleagues have shown that IFN-I induced the expression of several pro-apoptotic molecules such as Bim and Bax in pDCs, causing caspase activation and cell death [45]. Additionally, sustained IFN-I expression during persistent infections disrupts splenic architecture, which results in impaired immune cell interactions [26,29]. It also increases interleukin 10 (IL-10) and Programmed death-ligand 1 (PDL-1) expressions on resident immunosuppressive DCs, monocytes, and macrophages; as well as reduces total cell numbers of splenic DCs, macrophages, and natural killer cells [4,26,29,46]. This collective innate immune dysfunction is significantly rescued by IFNR blockade [26,29,46] (Table 1). Similarly, IFNR blockade in lymphocytic choriomeningitis virus clone 13 (LCMV clone 13)-infected mice reduces the levels of pro-inflammatory cytokine IL-1 and IL-18 in plasma, and decreases the expression of active caspase-1 in DCs and macrophages [26,29]. This indicates that IFNR blockade may counteract both inflammation and inflammasome activation in innate immune cells during persistent viral infection. The progressive loss of DCs due to sustained IFN-I exposure could be explained by the downregulation of microRNA221 and resultant expression of pro-apoptotic genes such as *bim* and *foxo3a* [47]. Cunningham et al. have recently shown that, in addition to induction of IL-10 and PDL-1 in immunosuppressive DCs, sustained IFN-I expression during LCMV clone 13 infection simultaneously inhibited the generation of cDCs with T-cell stimulating capacity [48]. Furthermore, Honke and colleagues revealed that enforced viral replication in marginal zone CD169^+^ macrophages, which is essential to ensure proper antigen synthesis, was blunted in infected mice in an IFN-I-related manner [23,49]. Interestingly, if blocking IFN-I increases viral replication, more antigen would be produced, potentially stimulating the adaptive immune response. Implementing this viral replication could be seen as a new “shock and kill” approach to current antiviral treatment. Sustained IFN-I expression during persistent viral infection promotes immunosuppression through the expansion and accumulation of Ly6C^high^ monocytes that are functionally similar to myeloid-derived suppressor cells (MDSC) found in cancers [50,51]. Finally, findings from Rempel and colleagues show that IFN-α reprograms the innate immune response of monocytes and desensitizes them to normally activating microbial factors during chronic unsuppressed HIV-1 infection [52].

Altogether, these results show that sustained IFN-I expression during persistent viral infection drives significant innate immune dysfunction, which is ultimately responsible for increased inflammation and immunosuppression, along with reduced antigen presentation. Moreover, the results strongly suggest that IFNR blockade may be effective towards improving the innate immune response during persistent viral infection.

## 4. Detrimental Role of Sustained IFN-I Expression on the Humoral Immune Response

Persistent viral infections, including HIV-1, are associated with progressive and profound perturbation of humoral immune response as characterized by: (i) progressive depletion of memory and virus-specific B-cells; (ii) impaired vaccine response; (iii) polyclonal activation of B-cells resulting in heightened production of total and non-specific Abs (aka. hypergammaglobulinemia; HGG); (iv) abnormal distribution of B-cell subpopulations; and (v) delayed appearance of neutralizing Abs [13,54,56,61,62].

**Figure 2:**
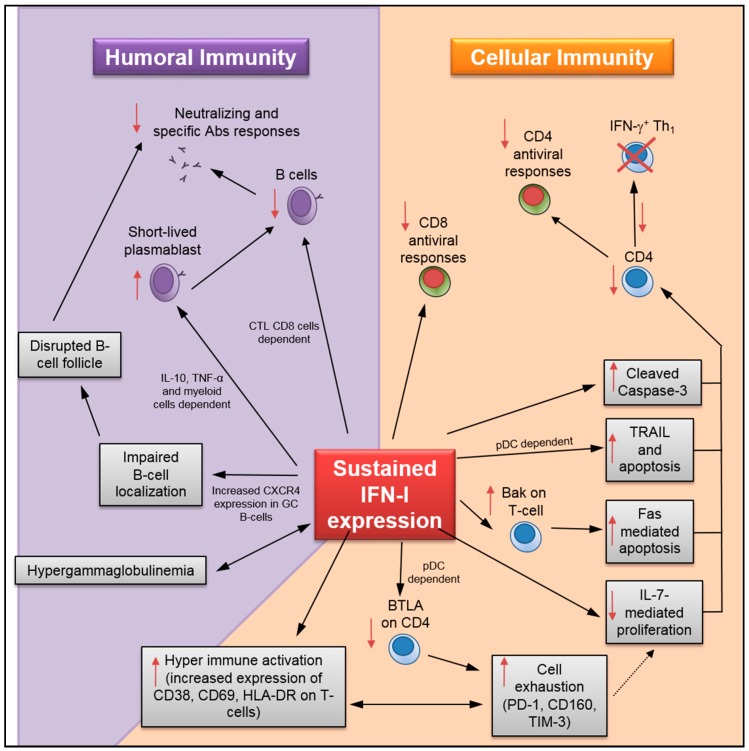
Detrimental effects of sustained IFN-I expression on humoral and cellular antiviral immunity. ↑: up-regulation; ↓: dow-regulation; 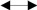: impact on both sides.

Recent evidence in LCMV clone 13-infected mice that were co-immunized with the 4-hydroxy-3-nitrophenylacetic (NP) hapten showed that sustained IFN-I expression was responsible for HGG as well as reduced production of NP-specific Abs [63]. In this study, Daugan et al. also observed the delayed appearance of LCMV-specific neutralizing Abs along with disrupted B-cell follicle structure and high expression of chemokine receptor CXCR4 in germinal center (GC) B-cells [63] (Figure 2). It is important to note that aberrant expression of this receptor affects the homing of B-cells within the follicles, which resultantly impacts the architecture of this microstructure. Hence the disrupted phenotype described above. Although IFNR blockade has no effect on HGG, the treatment resulted in significant restoration of the NP-specific response that was illustrated by higher levels of specific Abs and Ab-secreting cells (ASC), along with reduction of B-cell C-X-C motif chemokine receptor 4 (CXCR4) expression and partial recovery of B-cell follicles (Table 1). Taken together, these findings suggest that, in addition to improving antigen-specific Abs responses, IFNR blockade may likely prevent atypical B-cell trafficking and localization outside of follicles. Furthermore, the authors showed that the effects of IFN-I were observed in a B-cell intrinsic manner whereby prolonged exposure to the cytokine bore direct effects on the B-cells. As such, IFNR deficiency in B-cells accelerated the development of LCMV-specific neutralizing Abs. The control of LCMV infection by IFNR blockade has also been associated with increased number of splenic B-cells [26]. Finally, three recent studies have demonstrated the detrimental role of sustained IFN-I expression on the survival of specific B-cells, the generation of short-lived plasmablasts, and neutralizing Abs in LCMV clone 13-infected mice [55,57,58]. In these studies, defective humoral immune responses were not directly attributed to B-cell-intrinsic IFN-I sensing, but rather due to reduced antiviral B-cell immune response ascribed to other effectors such as cytotoxic T-lymphocyte CD8 T-cells (CTL), IL-10^+^ myeloid cells, and inflammatory monocytes via nitric oxide production [55,57,58]. Of note, despite the difference in direct versus indirect effect on B-cells, virus-specific humoral responses in the latter setting were rescued using IFNR blockade [55,57].

Overall, these data indicate that both direct and indirect effects of sustained IFN-I expression in B-cells contribute towards humoral dysfunction during persistent viral infection and can be counteracted by IFNR blockade.

## 5. Impact of Sustained IFN-I on T-Cell Maintenance and Antiviral Response

Chronic inflammation is a major hallmark of disease progression during persistent viral infection and, in the case of HIV-1 infection, is also observed in patients undergoing ART. This ultimately results in: (i) increased sensitivity to apoptosis and cell loss in CD4 population; (ii) T-cell hyperactivation; and (iii) exhaustion along with defective antiviral response [10,11,14,64,65].

(i) T-cell loss: Although IFN-α administration initially prevents systemic SIV infection in rhesus macaques, prolonged treatment accelerates CD4 T-cell loss [6]. A recent study by Chen and colleagues has shown that sustained IFN-I expression increased HIV-1-induced apoptosis and caspase-3 activity in CD4 T-cells [19] (Figure 2). Importantly, INFR blockade rescued HIV-specific T-cell and total T-cell numbers during persistent infection, as well as reduced the apoptosis in CD4 T-cells (Table 1). Herbeuval and colleagues have shown that IFNR blockade results in decreased TRAIL/DR5-mediated apoptosis and caspase-3 activity in CD4 T-cells using an in vitro HIV-1 infection model [53]. In a separate study, they also reported a lower frequency of TRAIL^+^ and apoptotic CD4 T-cells in HIV-1-infected human samples after treatment with anti-IFNα/β neutralizing Abs [59]. In addition, it has also been shown that IFNR blockade causes downregulation of cell-death signal cascades by the reduction in Bak expression and Fas-mediated apoptosis in CD4 T-cells using an in vitro HIV-1 infection model [66]. IFNR blockade also increases total splenic T-lymphocyte and antiviral specific CD4 T-cell numbers during LCMV_CL13_ infection [26,29,63]. Finally, data collected by Cha and colleagues indicates that sustained IFN-I expression in HIV-1-infected patients undergoing ART may promote T-cell loss by accelerating cell turnover and activation-induced cell death while decreasing T-cell homeostasis mediated by IL-7 [7,60]. Similarly, prolonged exposure to IFN-I in mice under lymphopenic conditions has been found to alter CD4 T-cell homeostasis [67].
(ii) T-cell hyperactivation: Elevated expression of T-cell activation/proliferation markers such as CD38, HLA-DR, and Ki67, which correlates with sustained IFN-I expression during persistent HIV-1 infection [8], is significantly reduced by IFNR blockade [18,38]. Similarly, INFR blockade reduces HIV-induced CD80 expression in CCR5^+^ T-cells, and CD69 and CD38 in T-cells during in vitro infection [46,68]. HIV-1-induced BTLA downregulation in T-cells, which may also contribute to hyperactivation, can be prevented by IFNR blockade [69].
(iii) T-cell exhaustion: During chronic HIV-1 infection, the IFN-I pathway is associated with CD4 T-cell exhaustion [70]. Recent results in humanized mice infected with HIV-1 have shown that IFNR blockade resulted in reduced expression of several exhaustion markers in CD8 T-cells—including PD-1, CD160, and TIM-3—along with enhanced IFN-γ and IL-2 production in virus-specific T-cells [18,19,38]. Relatedly, IFNR blockade in LCMV_CL13_-infected mice enhances virus-specific CD4 T-cell response [26,29]. Finally, sustained IFN-I expression during LCMV_CL13_ infection also suppresses de novo Th_1_ differentiation in late primed virus-specific CD4 [71]. In this study, the authors have shown that, although reduced Th_1_ differentiation was not mediated through direct IFN-I sensing by CD4 T-cells, it could be rescued by IFNR blockade.
(iv) Impact on immunosuppressive Treg: Although the effect of IFN-I signaling on hyperactivation and cell exhaustion is evident, its impact on regulatory T cells (Treg) during viral infections remains unclear [3]. In the case of acute LCMV infection, studies provide contradicting information showing either no effect of IFN-I on Treg [72], or a direct effect in reducing their numbers resulting in lower viral load [73]. Moreover, the effect of Treg depletion during chronic LCMV infection failed to increase viral clearance due to PD-L1 expression on infected cells despite an increase in virus-specific CD8 T-cell activity [74].

In summary, IFNR blockade during persistent viral infection decreases T-cell apoptosis, hyperactivation, and exhaustion, as well as improves antiviral immune response and cell maintenance.

## 6. Concluding Remarks

Overall, an increasing amount of evidence using human and in vivo models potentiates the beneficial outcomes of blocking IFN-I signaling during the chronic phase of viral infection once the viral persistence, chronic inflammation, and elevated IFN-I signatures are established in patients (Table 1). It is important to consider the complexity of the factors to ensure the safety and the clinical success for blocking IFN-I signaling-based therapy [3,28]. Namely, the timing of such treatment is critical considering that early administration of exogenous IFN-I is usually beneficial for the host and prevents the establishment of persistent infection [6,27,75] and, in the case of SIV, early IFNR blockade results in accelerated disease progression leading to AIDS [6]. As such, it is crucial to increase our knowledge on how sustained IFN-I signaling and timing of IFNR blockade precisely impacts molecular networks and immune cell phenotypes during persistent viral infections. This information will facilitate the translation of this therapeutic concept into successful treatment.

## Figures and Tables

**Table 1:** List of immune dysfunctions caused by sustained type I interferon (IFN-I) signaling which are reversibly rescued by the interferon-α/β receptor (IFNR) blockade.

Phenotype Improvement(s)	Model	Virus(es)	Reference(s)
Increased cytokine producing virus-specific CD4/CD8 (IFN-γ, TNF-α, IL-2); improved antiviral responses	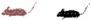	HIV, LCMV	[12,13,23,26,34]
Reduced expression of PD-1, TIM-3, TIGIT, BATF, CD160 on CD8 (decreased cell exhaustion)	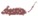	HIV	[12,34]
Reduced KI67^+^ population in CD4/CD8; decrease of HIV-mediated T-cell hyperactivation	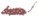	HIV	[12]
Reduced HLA-DR, CD38, CD69, CD80 expression in CD4/CD8; decrease of HIV-mediated T-cell hyperactivation	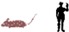	HIV	[12,34,41*,^53^*]
Decrease of caspase-3-dependent apoptosis in total and specific CD4 T-cells	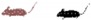	HIV, LCMV	[13,54,55*]
Accelerating neutralizing Abs production	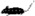	LCMV	[18]
Reduced PD-L1 and IL-10 expression in DCs, mono and macro. Decreased IL-10 levels in plasma/sera	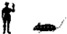	HIV, LCMV	[23,26,41*]
Reduced IL-1 and IL-18 levels, and inflammasome activation in DCs and monos; decreased CD80 expression in monos	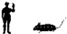	HIV, LCMV	[23,26,41*]
Increase of splenocyte cell numbers (DCs, macrophages, CD4, CD8, B and matural killers)	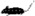	LCMV	[23,26,52]
Proper splenic architecture organization	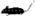	LCMV	[23,26]
Decreased CXCR4 expression on GC B Increased levels of specific Ab production and specific ASCs	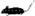	LCMV	[52]
Decreased caspase-3^+^ apoptotic virus-specific GC B (by counteracting plasmablast differentiation); B expansion	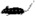	LCMV	[54]
Increased virus-specific B number; Decreased CTL CD8-mediated kill of specific B	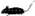	LCMV	[56]
Reduced TRAIL/DR5-mediated apoptosis in CD4	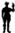	HIV	[55*]
Decreased % of TRAIL^+^ and apoptotic CD4	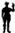	HIV	[57]
Increased Fas-mediated apoptosis in CD4 and CD8 T-cells	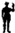	HIV	[58*]
Increased expression of BTLA on CD4 T-cells; reduced hyper-immune activation	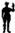	HIV	[59*]
Increased Th1 differentiation in late primed virus-specific CD4	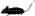	LCMV	[60]

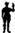
: Humans; 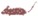: Humanized mice; 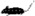; Mice; *: in vitro viral infection; ASC: antibody-secreting cells; CD: cluster of Differentiation; TNF: tumor Necrosis Factor; IL: interleukin; PD: programmed Death Protein; TIM: T-cell Immunoglobulin and Mucin; TIGIT: T-cell Ig and ITIM domain; BATF: basic leucine zipper transcription factor ATF-like; LCMV: Lymphocytic choriomeningitis virus; KI: proliferation Marker, **Ki**-67 is a prototype monoclonal Ab and was first produced in **Ki**el, Germany; HLA-DR: human leukocyte antigen - antigen D related; DCs: dendritic cells; CXCR: C-X-C motif chemokine receptor; GC: germinal center; ASCs: antibody secreting cells; CTL: cytotoxic T lymphocytes; TRAIL: TNF-related apoptosis inducing ligand; Th1: Type I T helper cells; BTLA: B and T lymphocyte attenuator.
